# Defining external factors that determine neuronal survival, apoptosis and necrosis during excitotoxic injury using a high content screening imaging platform

**DOI:** 10.1371/journal.pone.0188343

**Published:** 2017-11-16

**Authors:** Ujval Anilkumar, Petronela Weisova, Jasmin Schmid, Tytus Bernas, Heinrich J. Huber, Heiko Düssmann, Niamh M. C. Connolly, Jochen H. M. Prehn

**Affiliations:** Department of Physiology and Medical Physics and RCSI Centre for Systems Medicine, Royal College of Surgeons in Ireland, Dublin, Ireland; Universidad de Castilla-La Mancha, SPAIN

## Abstract

Cell death induced by excessive glutamate receptor overactivation, excitotoxicity, has been implicated in several acute and chronic neurological disorders. While numerous studies have demonstrated the contribution of biochemically and genetically activated cell death pathways in excitotoxic injury, the factors mediating passive, excitotoxic necrosis are less thoroughly investigated. To address this question, we developed a high content screening (HCS) based assay to collect high volumes of quantitative cellular imaging data and elucidated the effects of intrinsic and external factors on excitotoxic necrosis and apoptosis. The analysis workflow consisted of robust nuclei segmentation, tracking and a classification algorithm, which enabled automated analysis of large amounts of data to identify and quantify viable, apoptotic and necrotic neuronal populations. We show that mouse cerebellar granule neurons plated at low or high density underwent significantly increased necrosis compared to neurons seeded at medium density. Increased extracellular Ca^2+^ sensitized neurons to glutamate-induced excitotoxicity, but surprisingly potentiated cell death mainly through apoptosis. We also demonstrate that inhibition of various cell death signaling pathways (including inhibition of calpain, PARP and AMPK activation) primarily reduced excitotoxic apoptosis. Excitotoxic necrosis instead increased with low extracellular glucose availability. Our study is the first of its kind to establish and implement a HCS based assay to investigate the contribution of external and intrinsic factors to excitotoxic apoptosis and necrosis.

## Introduction

High content screening (HCS)-based assays can collect high volume, quantitative imaging data from complex cellular systems and enable the measurement of multiple features of cellular phenotypes relevant to the physiological and pathophysiological status of the cell. HCS was initially used primarily as a drug discovery tool to define the role of genes, proteins and biochemical pathways relevant to the therapeutic and toxic activities of drugs. However, in recent years HCS has emerged as a powerful tool to study protein function and the signaling pathways involved in cell signaling, survival and death [1–4]. However, there have been no HCS-based investigations of the signaling pathways involved in neuronal excitotoxicity.

The excessive activation of glutamate receptors, excitotoxicity, disrupts ion homeostasis and can lead to neuronal cell death, primarily due to massive Ca^2+^ deregulation [5]. Prolonged or severe activation of glutamate receptors induces irreversible disruption of ion homeostasis, mitochondrial bioenergetic status and cellular integrity leading to rapid necrotic cell death [6–10]. Conversely, transient or mild activation of glutamate receptors allows the cells to recover from the initial excitotoxic insult and instead induces delayed apoptotic cell death, characterised by collapse of mitochondrial bioenergetic status and delayed Ca^2+^ deregulation [10–13]. The neuronal ‘decision’ to survive, or undergo apoptotic or necrotic cell death in response to an excitotoxic challenge depends on the severity and duration of the insult [10,14]. The varying response of neuronal cultures to excitotoxic insults is also influenced by intrinsic factors, such as culture maturity and NMDA receptor composition [15–17], and external factors such as extracellular calcium [5,18] and glucose availability.

In this study we established, optimized and validated a HCS-based assay and analysis workflow to monitor post-excitotoxic nuclear morphology and plasma membrane integrity, respectively indicative of apoptosis and necrosis. We here show that this assay can be utilized to study the influence of external components involved in excitotoxic cell death such as neuronal plating density, and extracellular Ca^2+^ and glucose availability. In addition, by inhibiting key signaling pathways involved in neuronal apoptosis, we demonstrate that this HCS-based assay can also be utilized to investigate the role of intrinsic signaling components in neuronal fate decisions following excitotoxic injury. Cumulatively, this assay can be used as a high-throughput method to assess the interactions between external environmental factors and intrinsic signaling pathways in regulating neuronal survival, apoptosis and necrosis.

## Materials and methods

Fetal bovine serum, horse serum, minimal essential medium (MEM), Propidium Iodide and Hoechst 33342 were from Invitrogen (Bio Sciences, Dublin, Ireland). Poly-D-lysine pre-coated 96 well plates were from Corning (Flintshire, UK). Glutamate and all other chemicals were from Sigma (Wicklow, Ireland) and glycine was from Biomol (Hamburg, Germany). Pharmacological compounds are listed below.

### Preparation of mouse cerebellar granule neuron cultures

Mouse cerebellar granule neurons were prepared as described previously (Ward et al., 2000) with minor modifications. Briefly, cells were cultured on Poly-D-lysine pre-coated 96 well plates at a density of 50,000 cells/well (unless indicated otherwise) in 100 μl of culture media (minimal essential medium (MEM) supplemented with 10% (v/v) fetal calf serum, 20 mM KCl, 2 mM L-glutamine, 15 mM glucose unless stated otherwise, 50 units/mL penicillin and 50 μg/mL streptomycin). Neurons were maintained at 37°C in a humidified atmosphere of 5% CO_2_/95% air. The outer wells of each 96 well plate were filled with water to reduce possible edge effects. Experiments were carried out after 7–9 days *in vitro* (DIV). Given the short timeframe in which the characteristics of primary neurons remain consistent (DIV 7–9) and a 24 hour experiment, we utilized 2 wells per condition to maximize the number of conditions that we could assay, and repeated experiments on multiple plates. All animal work was carried out with ethics approval from the Royal College of Surgeons in Ireland Research Ethics Committee and under the licenses obtained from the Irish government granted to the authors under the Cruelty to Animals Act, 1976. The number of pups killed was recorded and annual reports were submitted to the Irish Department of Health and Children.

### Glutamate excitotoxicity

Cerebellar granule neurons (CGNs) were excited with glutamate/glycine at concentrations of 10, 30, 100 or 300 μM glutamate / 10 μM glycine for 10 or 30 minutes (unless stated otherwise) in experimental buffer composed of (in mM) 120 NaCl, 3.5 KCl, 0.4 KH_2_PO_4_, 5 NaHCO_3_, 20 HEPES, 1.2 Na_2_SO_4_ supplemented with 15 glucose and 1.2 CaCl_2_ at pH 7.4, although the glucose and calcium concentrations were varied in some experiments. Following treatment, cultures were washed with 1.2 mM MgCl_2_-supplemented experimental buffer to inhibit glutamate receptor activation and returned to preconditioned media containing Propidium Iodide, for high-content screening.

### Drug treatments

CGNs plated at 50,000 neurons/well were cultured for 7–9 DIV and were pre-treated for 1 h prior to glutamate excitation with a calpain inhibitor (Calpeptin, 20 nM), a mammalian target of rapamycin (mTOR) inhibitor (Rapamycin, 250 nM) or a poly (ADP-ribose) polymerase (PARP) inhibitor (DPQ, 100 μM) (see Table 1 for compounds used in this study). Following glutamate exposure, media was replaced with preconditioned media containing the inhibitors described above and imaged for 24 h on the HCS platform. CGNs were only pre-treated for 1 h prior to glutamate excitation with AMPK inhibitor (Compound C, 10 μM) or c-Jun N-terminal Kinases (JNK) inhibitor (SP600125, 5 μM). Following glutamate exposure, media was replaced with preconditioned media without the inhibitors. In the assay development phase, neurons were treated with 300 nM staurosporine (STS) for 24 h prior to imaging to induce robust apoptosis [19,20], or 0.01% Triton-X-100 for 10 min prior to imaging to induce plasma membrane rupture as an indicator of necrosis.

**Table 1:** List of compounds used in this study.

Compound	Solubility	Catalogue No	Manufacturer
Compound C	DMSO	171261	Calbiochem
Calpeptin	DMSO	ALX-260-014	Alexis biochemicals
SP 600125	DMSO	EI305	Biomol
DPQ	DMSO	D5314	Sigma-Aldrich
Rapamycin	DMSO	9904	Cell Signaling

### High content screening: Imaging

CGNs were stained with Hoechst (100 ng/ml) for 1 h and Propidium Iodide (PI) (250 ng/ml) was present in the preconditioned media throughout imaging. Neurons were imaged in 96 well plates using a Cellomics ArrayScan VTi instrument (Thermofisher, Surrey, UK) equipped with a 10x PlanApo objective lens (NA 0.45), 120 W Hg arc illumination source (EXFO) and a monochrome CCD camera (Hamamatsu Orca AG). Time series of images (9 fields of view) were collected from each well (total of 5,000–6,000 neurons when seeded at 50,000 cells/well) at 1 h intervals, with 1024x1024 pixel resolution (645 nm pixel size). Dye concentration and image acquisition rate were optimized to reduce phototoxicity. PI was excited at 545–575 nm and emission was collected through a 590–625 nm band-pass filter. Hoechst was excited at 381–394 nm and emission light was collected through a 415–460 nm band-pass filter. Fixed exposure parameters were used and varied between the experiments from 0.6–2.0 s for Hoechst and 0.5–1.0 s for PI. The autofocus module on the Cellomics Array scanner was applied in alternating fields to keep the neurons in focus. The focus was fixed on the PI channel to avoid phototoxicity caused by focusing on the Hoechst channel.

#### High content screening analysis workflow: Background correction and segmentation (Fig 1Ai)

**Fig 1:**
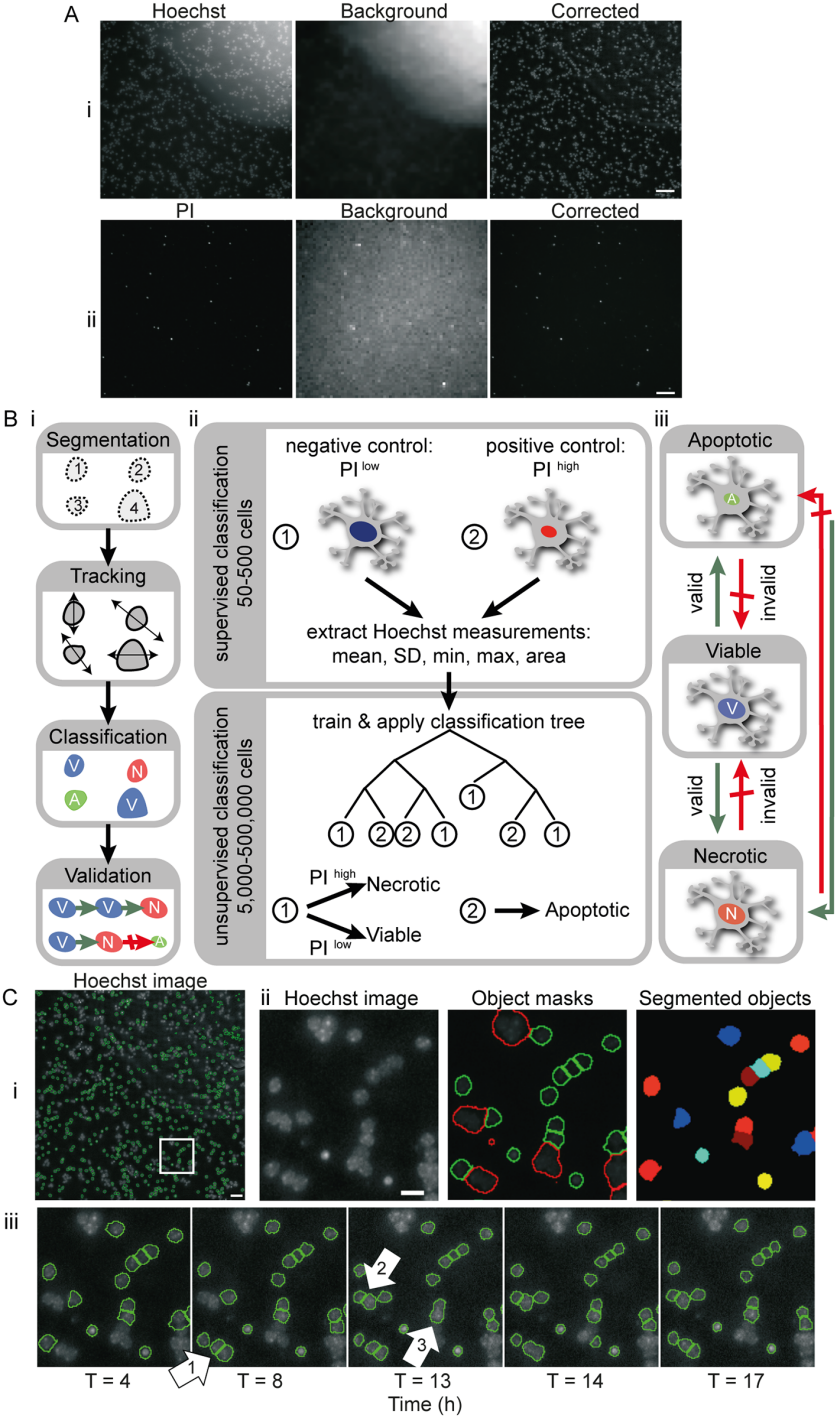
High content screening (HCS) workflow: Background correction, segmentation and classification of Hoechst and PI stained neurons in real time HCS experiments. A) Representative images of mouse cerebellar granular neurons cultured in 96 well plates and stained with (i) Hoechst (100 ng/ml for 1 h) and (ii) PI (250 ng/ml). Variations in background signal due to illumination fluctuation were corrected using CellProfiler. Scale bar: 40 μm. B) Time-lapse images obtained from HCS experiments were processed using CellProfiler and MATLAB. i) Flow chart showing steps involved in analysis of stained neurons following image acquisition on the HCS system—nuclei segmentation and measuring, tracking objects over time, and classification and validation of time-lapse images from HCS. ii) Representative cells were selected for classification by a human expert—nuclei characteristics were extracted and used as a training set for unsupervised classification by binary decision tree. Nuclei were categorised as: Class 1 –when the Hoechst area was large and uniform with even staining; or Class 2 –apoptotic cells showing condensed nuclei and intense Hoechst staining. Based on PI fluorescence intensity, objects in Class 1 were further distinguished as viable (low PI) and necrotic (high PI) neurons. iii) Changes in classification of each cell over time were tested and validated. Green and red arrows indicate valid and invalid state transitions, respectively. C) Neurons were segmented using a combination of Otsu thresholding and watershed algorithm, implemented in CellProfiler. (i) Representative Hoechst image of a field of view acquired by HCS, showing masks for Hoechst stained nuclei (green outlines). Scale bar: 40 μm. (ii) Representative Hoechst image (zoomed in white box in i), object masks and segmented object areas from CellProfiler. Green masks represent objects that were measured for further analysis while objects showing red outlines were discarded due to size restrictions, mostly caused by under-segmentation of clumped cells. Scale bar: 10 μm. (iii) Time lapse image series of Hoechst stained neurons showing variation in segmentation. Annotation (white arrow) 1 & 2 show identification and segmentation of previously unsegmented neurons. Annotation 3 shows inaccurate object segmentation which had been correctly segmented in previous timepoints.

All images were segmented and measured using CellProfiler (Broad Institute, Cambridge, Massachusetts, USA). CellProfiler provides the capability to measure the size, shape and fluorescence intensity of cells, and is purpose built for standardized quantitative analysis of high-throughput and high content imaging, with structured processing and analysis modules available (Carpenter et al., 2006). In this study, we optimized and combined modules for uneven/non-uniform background illumination correction, nuclei isolation and shape/intensity parameter identification to create a HCS image processing and analysis workflow.

Non-uniformities in background fluorescence (Fig 1Ai) are likely due to imperfections in plate bottom and/or non-specific dye binding. To overcome this challenge, the “CorrectIlluminationCalculation” and “CorrectIlluminationApply” modules were applied to correct the images for non-uniform background illumination (Fig 1Ai), and to calculate an effective background function for images that seem uniformly illuminated (Fig 1Aii). As any lamp fluctuations in the foreground and background of the image originate from the same source, the illumination function was calculated on the background and then subtracted from the original image. Each image was split into square blocks of 8x8 pixels to find the minimum pixel intensities of background in blocks across each image for non-uniform illumination. The block size was determined such that each block was likely to contain sufficient background pixels. A median filter was then applied to these blocks to achieve a smooth illumination pattern. The advantage of the median filter is its robustness against outliers such as very high fluorescence intensities of cell debris or dust particles. The size of the smoothing filter is automatically set by the software based on estimated object size of the artifacts to be corrected.

The module “IdentifyPrimaryObjects” utilized the size and intensity range of the stained nuclei to distinguish areas of interest from background and noise, with its parameters optimized for Hoechst staining and PI staining individually. A combination of the Otsu adaptive threshold method and a watershed algorithm as provided by CellProfiler was best suited for segmentation of nuclei. The Otsu method determines a threshold to distinguish foreground from background signal (Fig 1Cii). We noted that this adaptive method is more robust towards bright and dim regions within a Hoechst-stained nucleus. However, this resulted in clumped cells sometimes being under-segmented as a single object if there was no area below the determined threshold between the cells (see Fig 1Ciii, annotation 3). This was improved using a watershed algorithm based on shape and smoothing with a filter size of 5 pixels. Additionally, a defined threshold for the distance between local intensity maxima was used to avoid over-segmentation. The nuclei of Hoechst-stained CGNs are between 9 and 20 pixels in size, while the signal of PI staining covers an average area of 7–20 pixels per object. Identified object masks smaller or larger than these ranges were discarded from the set of segmented objects (red object masks in Fig 1Cii). Accurate segmentation is typically achieved for PI stained nuclei since the condensed nuclei are at sufficient distance from neighboring signals and the fluorescence intensity is strongest in the center and dim towards the edges (S1 Fig). The resulting measurements generated from CellProfiler (object shape, size and intensity) were imported to MATLAB (MathWorks^®^, UK) for tracking, classification and validation.

#### High content screening analysis workflow: Tracking and classification

For image data analysis, we employed MATLAB with the freely available tracking algorithm u-track [21]. We modified and adapted the u-track software to allow us to track CGNs in time-lapse imaging. Despite our best efforts, segmentation in CellProfiler was not accurate. For example, in Fig 1Ciii, fluctuations are apparent in a number of objects over time, due to under-/over-segmentation, discarded masks due to size limits, and cells not detected or moving in/out of view. We therefore used u-track to recover information by tracking measurements over time: 1) Closing gaps helped for objects lost temporarily (maximum allowed gap length in tracks was limited to 3 frames, otherwise track was discarded). 2) Splitting and merging tracks over time, depending on the objects detected between the consecutive frames, helped to overcome issues with under-/over-segmentation. 3) When objects appeared only at later timeframes (due to fluctuations in image acquisition), or disappeared towards the end of the experiment (most likely due to cell death), we extended the track classification to the start/end of the experiment.

The classification of nuclei into apoptotic, necrotic and viable cells was achieved by a combination of supervised and unsupervised multiparameter classification (Fig 1Bii). PI intensity, nuclear area, and mean, minimum, maximum and standard deviation of the Hoechst intensity were extracted from the representative images and used to classify the CGNs as follows:

1. Viable: Neurons with large, uniform nuclei, even distribution of Hoechst, and low PI intensity.
2. Apoptotic: Neurons with condensed nuclei and increased Hoechst intensity, but low PI intensity.
3. Necrotic: Neurons with large, uniform nuclei and high PI intensity.

A human expert performed a supervised classification of a small set of cells. Based on experimental conditions and visual nuclei characteristics, nuclei were categorized as condensed (apoptotic) or as a group showing large uniform nuclei (viable or necrotic). From this set, Hoechst staining characteristics (area, mean, minimum, maximum, standard deviation) were extracted and used to train a binary classification tree as implemented by MATLAB. Following unsupervised classification by the trained decision tree, a PI threshold was determined from representative images to further distinguish viable from necrotic cells. The classifications at each time point were regulated by allowing only physiologically feasible transitions to occur (Fig 1Biii, green arrows). The classification history was calculated both forwards and backwards and the track corresponding to minimum discrepancy was chosen. The validation of this classification algorithm is described in the Results.

### Descriptive statistical and data analysis

Significant differences between medians and means were determined by Wilcoxon rank sum and one-way ANOVA followed by post-hoc Tukey’s test, respectively, and corrected for multiple comparisons (Bonferroni). Traces, boxplots and heat maps were generated in MATLAB R2016b. In all figures, heatmaps show the median value of all wells from the indicated treatment. Time-course traces plot the median (dark lines) surrounded by the (shaded) inter-quartile regions over 24 h. Boxplots have lines at the lower, median, and upper quartile values, and the whiskers extend to the most extreme value within 1.5 times the interquartile range. Crosses indicate wells outside this range. Each individual data point, plotted next to the boxplots, corresponds to the value from one well.

## Results

### Validation of a HCS imaging analysis workflow to monitor neuronal apoptosis and necrosis

To develop a HCS platform to monitor neuronal viability, apoptosis and necrosis, mouse CGNs cultured in 96 well plates and stained with Hoechst and PI were imaged for 24 h using a Cellomics ArrayScan VTi instrument (Thermofisher, Surrey, UK). An imaging analysis workflow was built in CellProfiler and MATLAB, consisting of sequential modules specifically optimized for the processing and analysis of our HCS images. The workflow comprises modules for background correction, object segmentation, cell tracking and classification of segmented objects (Fig 1B).

**Fig 2:**
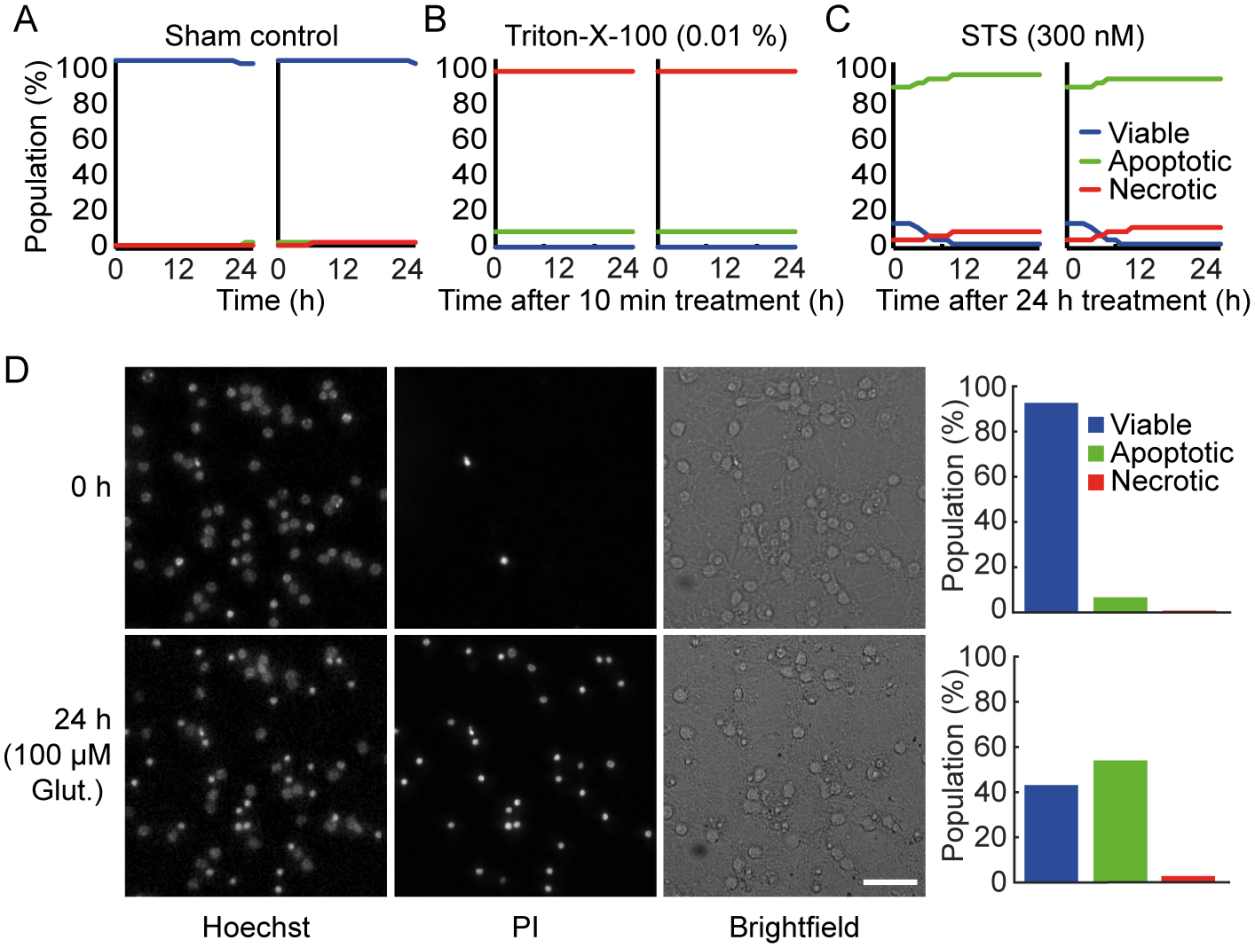
Classification and validation of viable, necrotic and apoptotic population in CGNs. A) The high content screening workflow detected mostly viable cells (blue traces) when neurons were treated with control media. B) Treating wells with 0.01% Triton-x-100 for 10 min to induce necrosis prior to imaging resulted in most cells being classified as necrotic (red traces). C) Treating wells with the apoptosis inducing staurosporine (STS) at 30 nM concentration for 24 h prior to the start of image acquisition, resulted in cells primarily being classified as apoptotic (green traces). Each graph represents 9 fields of view (550–650 cells / field of view), taken from each well for 24 h with 1 h time interval between each scan. Graphs are representative of 2 independent experiments. Two individual wells are shown to demonstrate the consistency of classification across wells. D) Representative Hoechst, PI and brightfield images at 0 h and 24 h following 100 μM glutamate treatment for 10 min. Quantification of viable, apoptotic and necrotic populations in these wells (bar charts) demonstrated that neuronal cell death 24 h after transient glutamate exposure was primarily apoptotic.

Changes in nuclear morphology and cell membrane integrity were monitored using Hoechst and PI respectively, and used to classify CGNs as viable, apoptotic and necrotic (Figs 1Biii and 2). Hoechst stained cells showing regular nuclei with even distribution of the dye were classified as viable (Fig 2A, blue traces) and condensed nuclei with increased Hoechst intensity were classified as apoptotic (green traces). PI is taken up into the cell when the plasma membrane ruptures—a primary indicator of necrosis (red traces). In order to validate that our segmentation and classification parameters accurately predict the viable, apoptotic and necrotic populations in our cultures we performed control experiments with Triton-X-100, which causes plasma membrane rupture and induces necrosis, and staurosporine (STS), a potent activator of apoptosis [19]. Neurons treated with 0.01% Triton-X-100 for 10 min were primarily classified as necrotic (Fig 2B), while neurons treated with STS (300 nM) for 24 h prior to image acquisition showed a significant increase in the apoptotic population (Fig 2C). This indicated that the classification parameters were optimal to distinguish between viable, apoptotic and necrotic populations in our system. Using the classification parameters described above, we were able to accurately distinguish between viable, apoptotic and necrotic populations in neurons exposed to transient glutamate excitotoxicity (Fig 2D).

### Neurons seeded either at low or high density undergo increased necrosis

**Fig 3:**
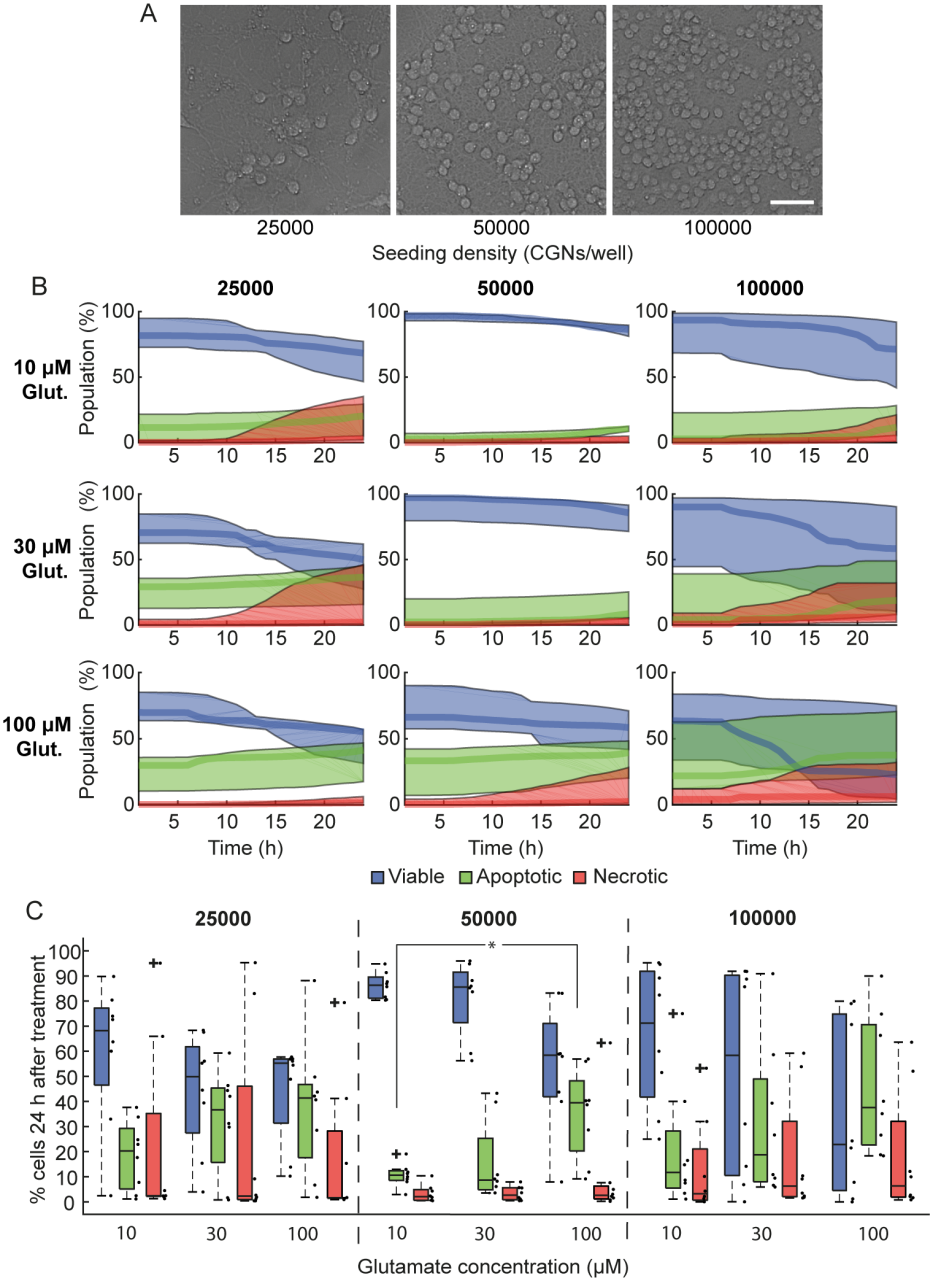
CGNs seeded either at low or high seeding densities undergo increased necrosis in response to glutamate excitation. Cerebellar granule neurons (CGNs) seeded at 25,000, 50,000 or 100,000 cells/well were cultured for 7/8 DIV and treated with 10, 30 or 100 μM glutamate (Glut; with 10 μM glycine) for 10 min before media was replaced with high Mg^2+^ (1.2 mM) containing buffer to block glutamate receptors. For 24 h following glutamate exposure, 9 fields of view per well (550–650 cells/field of view) were imaged at 1 hr intervals, and neurons were classified as viable, apoptotic or necrotic based on their nuclear morphology (Hoechst) and plasma membrane integrity (PI). A) Representative transmitted light images of CGNs seeded at 25,000, 50,000 or 100,000 cells/well in 96-well plates and cultured *in vitro* for 7 days. Scale bar 40 μm. B) The population of cells (%), for each density and glutamate treatment, classified as viable (blue traces), apoptotic (green traces) or necrotic (red traces). Traces are median ± inter-quartile regions of all wells exposed to the same treatment. C) Quantification of viable, apoptotic and necrotic populations 24 h following glutamate exposure for each of the seeding densities and glutamate concentrations. Boxplots show the median ± inter-quartile regions (n = 8 wells for each treatment, from 4 independent experiments). Neurons seeded at 50,000 cells/well had lower well-to-well variability than neurons seeded at 25,000 or 100,000 cells/well, and underwent less necrotic cell death in response to glutamate excitotoxicity. Neurons seeded at 50,000 cells/well and treated with 100 μM glutamate underwent significantly increased apoptosis compared to neurons treated with 10 μM glutamate (*p = 0.014).

It has been previously demonstrated that overactivation of glutamate receptors can induce either apoptosis or necrosis depending on the severity and duration of the stimulus [10,14,22]. Seeding density may also impact neuronal physiology [23] and therefore the response to glutamate excitotoxicity. HCS screening platforms can simultaneously investigate multiple conditions in single experiments. We utilized our HCS-based imaging and analysis workflow to investigate the response of CGNs seeded at different densities to transient exposure to glutamate at different concentrations. CGNs were seeded at different densities (25,000, 50,000 or 100,000 neurons / well) (Fig 3A) and stimulated with 10, 30 or 100 μM glutamate / 10 μM glycine for 10 minutes. Neurons plated at 50,000 neurons/well primarily underwent apoptotic cell death in response to glutamate-induced excitotoxicity, while neurons cultured at either low or high- density (25,000 or 100,000) underwent increased levels of necrosis (Fig 3B and 3C). Interestingly, neurons plated at 50,000 cells/well also exhibited lower well-to-well variability than other seeding densities, and showed a significant increase in apoptotic cell death when treated with 100 μM glutamate compared to 10 μM (Fig 3C, p = 0.014).

### Increased extracellular Ca^2+^ induces cell death mainly through apoptosis

**Fig 4:**
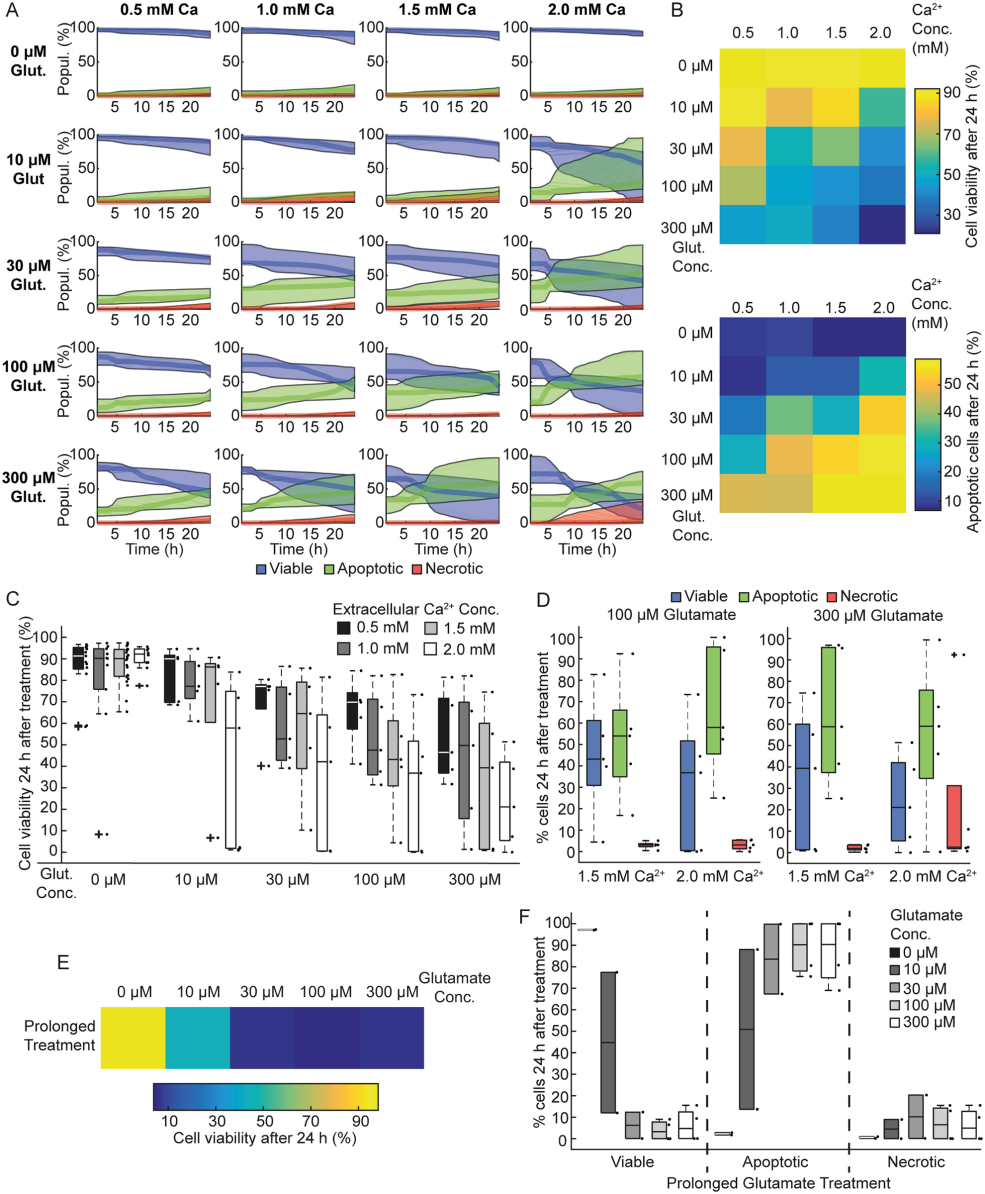
Increased extracellular Ca^2+^ concentration sensitizes CGNs to excitotoxic insult and induces cell death mainly through apoptosis. CGNs were seeded at 50,000 cells/well and cultured *in vitro* for 7/8 days. CGNs were treated with different glutamate (Glut.) and Ca^2+^ concentrations as indicated for 10 min. A) The population (Popul.) of CGNs (%), for each glutamate and Ca^2+^ concentration, classified as viable (blue traces), apoptotic (green traces) or necrotic (red traces). Traces shown are median ± inter-quartile regions. Neurons exposed to high glutamate and high Ca^2+^ were more sensitive to cell death. B) A heatmap of median cell viability 24 h following glutamate exposure illustrates lower viability in CGNs exposed to increasing glutamate and extracellular Ca^2+^ concentrations. C) Populations of viable cells 24 h following glutamate excitation. Neurons exposed to 2.0 mM Ca^2+^ (white boxes) were more vulnerable to glutamate excitation. Boxplots show the median ± inter-quartile regions (n = 10 wells for each treatment, from 5 independent experiments). D) Populations of viable, apoptotic and necrotic neurons 24 h following exposure to 100μM or 300μM glutamate at 1.5 and 2.0 mM extracellular Ca^2+^. Neurons exposed to 300 μM glutamate and 2.0 mM Ca^2+^ show increased necrotic cell death. E) Heat map illustrating prolonged glutamate exposure induced cell death at 24 h post glutamate excitation. F) Distribution of viable apoptotic and necrotic neurons in response to prolonged glutamate excitation (100 μM /10 μM glycine) at 1h and 24 h post excitation. Quantification from 4 wells from 2 independent experiments. Boxplots show the median ± inter-quartile regions. Boxplot shows increase in apoptotic population in response to prolonged glutamate excitation. Total 8 wells from 4 independent experiments.

Several studies have shown that non-physiological increases in cytoplasmic Ca^2+^ levels during excitotoxic injury trigger various biochemical pathways such as endonuclease, calpain and caspase activation and ion transporter disruption, leading to apoptotic or necrotic cell death [24]. We wondered whether increasing external glutamate and/or external Ca^2+^ concentrations might induce necrosis in CGNs (Fig 4A, upper heatmap). Increased Ca^2+^ concentration was associated with increased cell death at all glutamate concentrations (Fig 4B). This is not surprising given the pivotal role of Ca^2+^ in executing neuronal death following excitotoxicity [5]. Indeed, we also measured elevated cytosolic Ca^2+^ in single cells incubated in increasing levels of extracellular Ca^2+^ and exposed to a transient glutamate insult (S3 Fig). Interestingly, this Ca^2+^-dependent increase in cell death was primarily due to apoptosis, as measured by our HCS workflow (Fig 4A and 4B lower heatmap). Higher levels of apoptosis were also seen with increasing glutamate concentration (Fig 4B). In addition, increasing both glutamate and Ca^2+^ together induced higher levels of cell death (Fig 4B and 4C), although only low levels of necrosis were observed (Fig 4D). To further characterize neuronal death in our system, we exposed CGNs to prolonged overexcitation of glutamate receptors, at varying concentrations (Fig 4E). Prolonged glutamate exposure was associated with increased neuronal death (Fig 4E). However, it failed to induce any necrotic death (Fig 4F) suggesting that, in our system, neuronal death in response to both transient and prolonged glutamate exposure is primarily mediated by apoptosis.

### Glucose availability sensitizes neurons to glutamate-induced excitotoxicity

**Fig 5:**
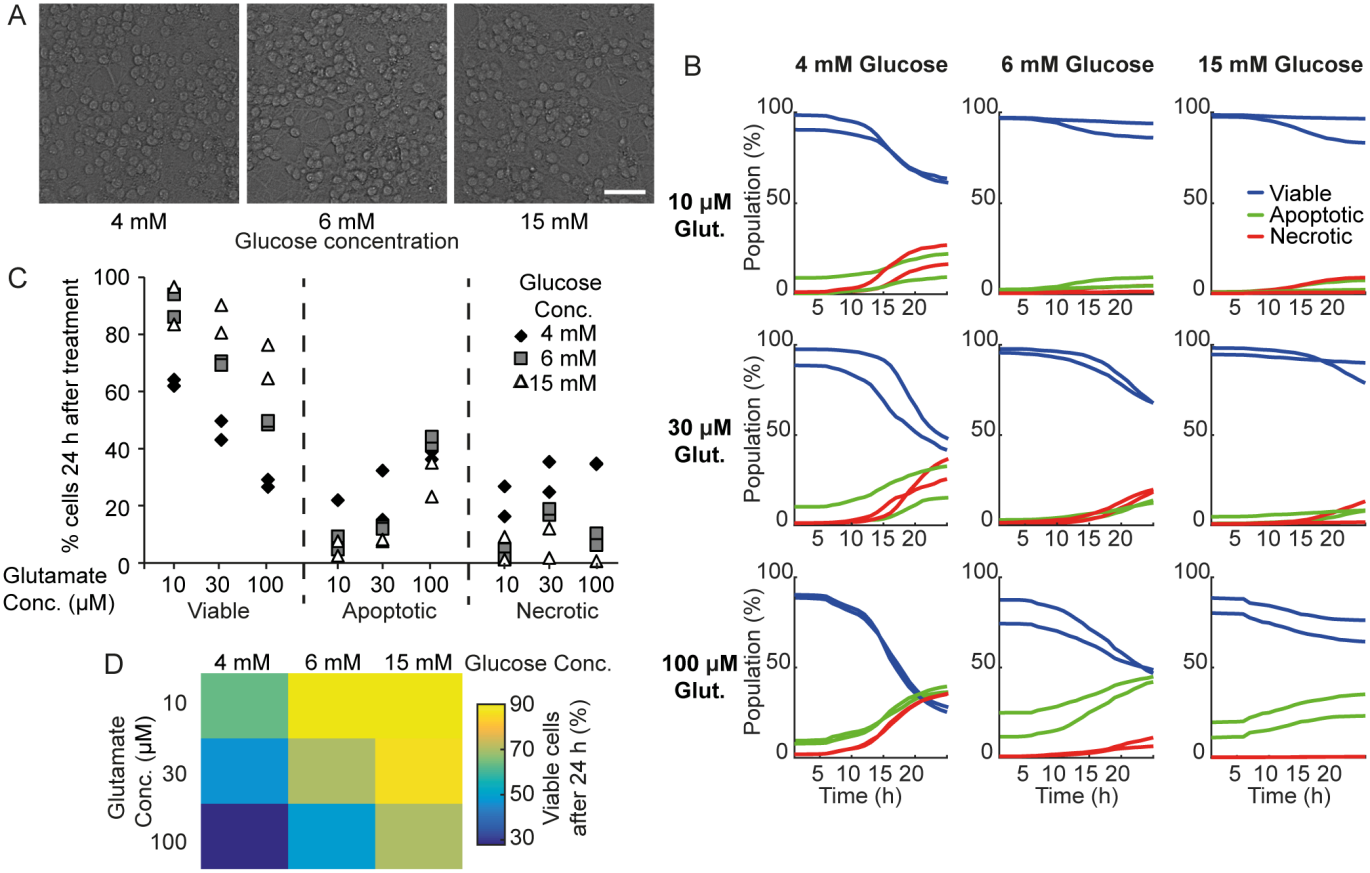
Decreased glucose availability increased sensitivity of neurons to glutamate excitation. Neurons seeded at 100,000 cells/well were cultured for 7 DIV in 4 mM, 6 mM or 15 mM glucose and treated with 10, 30 or 100 μM glutamate (Glut.), as indicated, for 10 minutes. A) Representative brightfield images showing that neurons cultured in lower glucose did not affect basal cell viability. B) The population of neurons (%) classified as viable (blue traces), apoptotic (green traces) and necrotic (red traces). Median traces are shown from two wells for each treatment. Neurons cultured in low glucose conditions are more sensitive to glutamate excitation. C) The population (%) of viable, apoptotic and necrotic neurons in each well 24 h following glutamate excitation. Neurons cultured in 4 mM glucose underwent increased necrosis. D) A heat map showing the median population of viable cells 24 h following glutamate exposure.

Glucose is an essential metabolic substrate for neurons and its delivery to neurons is dependent on the uninterrupted supply of glucose from the blood, as the ability of the brain to store and produce glucose is very limited. We next investigated the influence of extracellular glucose availability on the response of cultured CGNs to glutamate exposure of varying severity. Lower extracellular glucose concentrations reflect physiological levels measured *in vivo* [25], and previous studies showed that lower glucose concentrations in culture significantly decreased mitochondrial bioenergetic function and sensitized neurons to NMDA-mediated excitotoxicity, increasing necrosis [26,27]. CGNs seeded at 100,000 cells / well were cultured in medium containing 4, 6 or 15 mM glucose for 7 DIV. Lower glucose concentrations did not affect basal cell viability (Fig 5A). Neurons were treated with 10, 30 or 100 μM glutamate/10 μM glycine for 10 min in experimental buffer containing 4, 6 or 15 mM glucose. We first confirmed that no coordinated cell death was detected in this experiment, as they were plated at a higher density (data not shown). For all glucose concentrations, we again observed increased cell death with increasing glutamate concentrations (Fig 5B and 5C). Interestingly, neurons cultured in 4 mM glucose were more susceptible to all glutamate concentrations, and underwent higher levels of necrosis (Fig 5B and 5C). This could be due to lack of glucose availability leading to rapid and irreversible energy depletion. Neurons cultured in 6 mM glucose also exhibited higher cell death following 100 μM glutamate than neurons cultured in 15 mM glucose (Fig 5D). Interestingly, however, the neuronal death observed at 6 and 15 mM glucose concentration was primarily mediated through apoptosis (Fig 5C). Taken together, these results suggested that glucose availability plays an important role in sensitizing neurons to excitotoxicity, and that neurons cultured in moderate glucose concentrations (≥ 6 mM) primarily undergo excitotoxicity-mediated apoptotic cell death.

### Pharmacological inhibition of key cellular signaling pathways protects neurons against glutamate induced excitotoxicity

**Fig 6:**
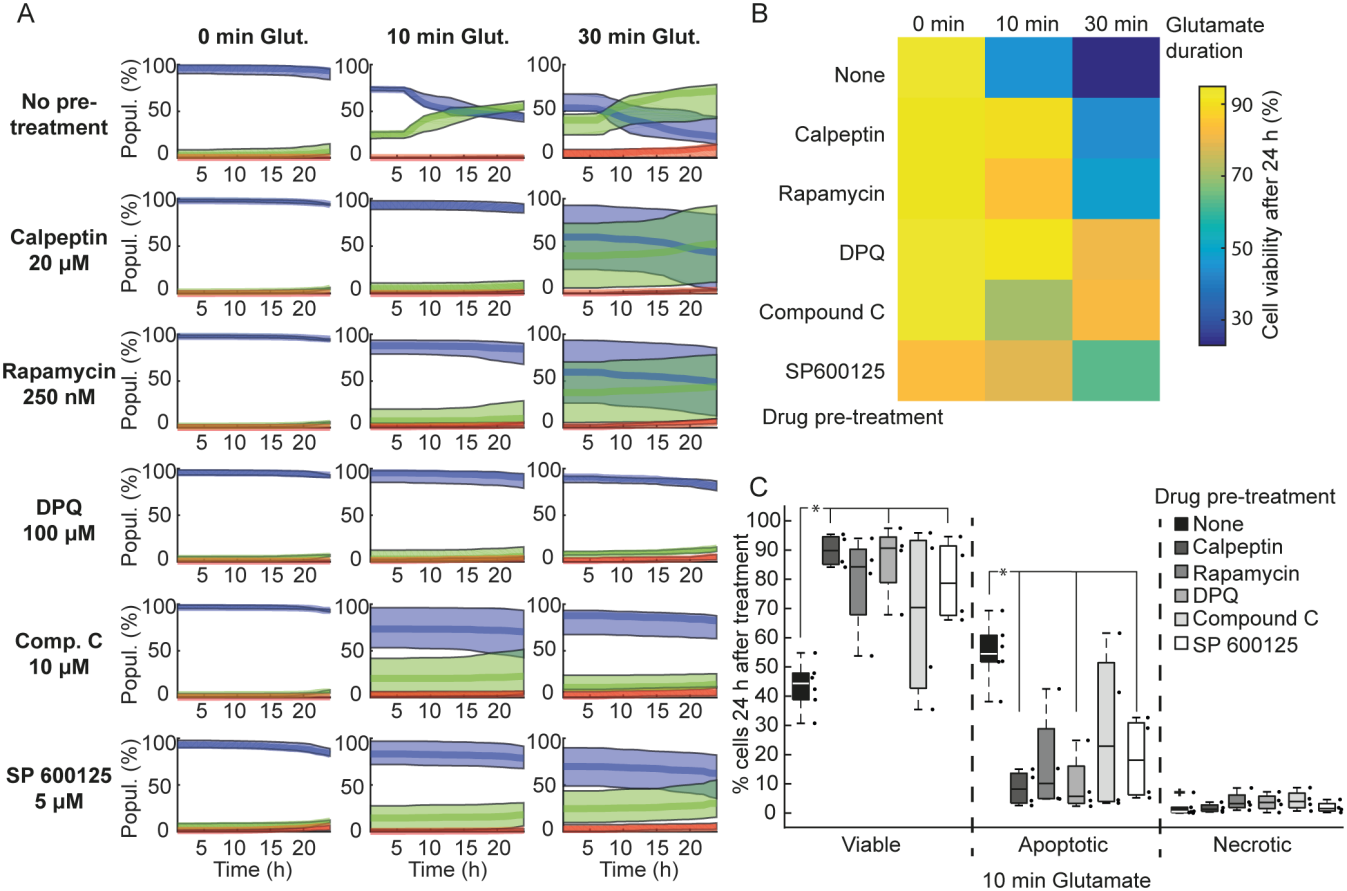
Pharmacological inhibition of cell death pathways protects neurons against glutamate-induced cell death. Neurons were seeded at 50,000 cells/well and cultured *in vitro* for 7/8 days. Neurons were pre-treated for 1h prior to glutamate excitation with calpeptin (20 nM), rapamycin (250 nM), DPQ (100 μM), Compound C (10 μM) or SP600125 (5 μM). Following glutamate exposure (Glut.; 100 μM for 10 or 30 min,), media was replaced with pre-conditioned media alone (in the case of Compound C and SP600125) or pre-conditioned media containing the drugs, and images were acquired for 24 h. A) Populations (Popul.) of neurons classified as viable (blue traces), apoptotic (green traces) and necrotic (red traces). Traces show the median ± inter-quartile regions (n = 4 wells from 2 independent experiments). Traces show that blocking key cell death signalling pathways protected neurons against excitotoxicity. B) A heat map of the median population of viable cells 24 h following glutamate excitation (100 μM) illustrates the protective effect of these drugs against glutamate-induced cell death. C) Boxplots showing % of viable, apoptotic and necrotic neurons 24 h following 100 μM glutamate excitation for 10 min. Boxplots show the median ± inter-quartile regions. Pre-treatment with Calpeptin, DPQ and SP 600125 significantly increased viability compared to no pre-treatment and this increased viability was primarily due to a decrease in apoptosis (*p = 0.0475 for all marked treatments compared to no pre-treatment).

We next used the high-throughput neuronal excitotoxicity model established in this study to assess the mechanisms of glutamate-induced neuronal apoptosis by blocking key signaling pathways shown to be involved in these processes (Fig 6A). The HCS assay enables the manipulation and investigation of multiple signaling pathways in a single experiment, through the application of various pharmacological compounds to different wells on a multi-well plate. In an effort to prevent neuronal apoptosis, the primary form of cell death in our system, we pre-treated CGNs with various pharmacological inhibitors as described below. It has been shown that calpains are activated during excitotoxic apoptosis, independent of caspase activation [11]. In addition, calpain activation occurs selectively during neuronal apoptosis, as there was no detectable activation in necrotic and tolerant neuronal population following excitotoxicity [14]. Here, we observed that treatment with calpeptin (a calpain inhibitor) significantly decreased apoptosis in CGNs transiently exposed to glutamate (Fig 6B and 6C, p = 0.0475). Next, we used rapamycin, an inhibitor of mammalian target of rapamycin (mTOR) implicated in various neurodegenerative diseases [28]. We again observed a marked increase in viability and decrease in neuronal apoptosis, although this decrease did not reach significance (Fig 6B and 6C). AMP-activated protein kinase (AMPK) plays a pivotal role in neuronal fate decisions. Chronic overactivation of AMPK induces neuronal death [29,30] whereas transient activation induces neuroprotection [31]. We treated neurons with Compound C to assess whether AMPK inhibition would improve neuronal survival following excitotoxicity. Indeed, Compound C decreased apoptosis in CGNs exposed to glutamate for varying durations, although not in all wells (Fig 6B and 6C). Furthermore, inhibition of the stress-related c-Jun N-terminal Kinases (JNK) using SP600125 also significantly increased neuronal resistance to excitotoxicity, while decreasing apoptosis (Fig 6B and 6C, p = 0.0475). Finally, we also investigated the role of the poly (ADP-ribose) polymerase (PARP) pathway, which induces rapid necrosis (parthanatos) in excitotoxicity [32,33]. Pre-treatment with a PARP inhibitor (DPQ, 100 μM) potently attenuated neuronal apoptosis following excitotoxic insult in CGNs (Fig 6C, p = 0.0475). Taken together, these results suggested that multiple cell death signaling pathways are involved in glutamate-induced apoptosis observed in our system, and that the HCS assay developed here can be employed to analyze kinetics of neuronal apoptosis over time. This assay could be used as a tool for drug screens and to identify therapeutic targets for various neurodegenerative diseases.

## Discussion

In this study we have developed and validated a practical, *in vitro*, cell-based High Content Screening (HCS) assay to assess neuronal apoptosis and necrosis following glutamate excitotoxicity. We developed a robust nuclei segmentation, tracking and classification workflow to distinguish between different neuronal fates. We based the tracking algorithm on u-track, a collection of functions developed to track fluorescently labeled intracellular proteins [21]. These functions also allow the closing of time-series data gaps, and for merging and splitting of objects created due to protein-protein interactions. In our case, gaps may arise because of deficient image acquisition (e.g. loss of focus, or artifacts hampering background correction) or failed segmentation. Merging and splitting data tracks overcome information loss due to under-segmentation of clumped cells. This high-throughput imaging and analysis workflow enabled information retrieval from many more neurons than would be possible manually. Together, the workflow allowed us to automatically and accurately track and categorize thousands of neurons as viable, apoptotic or necrotic over the entire course of the imaging experiment (24 h).

We showed that neurons seeded at optimal plating density (50k cells/well) mainly undergo apoptotic cell death in response to transient glutamate excitotoxicity, but that neurons seeded at either low or high density underwent increased necrosis. This may be due to aberrant neural network formation at sub-optimal plating densities [23]. In this context, neurons cultured at high densities increase their glutamate release [34], potentiating the excitotoxic extracellular environment. This may also help to explain the coordinated cell death observed in neurons plated at high density in our system (S2 Fig). In this pattern, neuronal death starts at one point in the field of view and moves in a wave throughout the field of view until all neurons are dead (S1 Movie). Coordinated cell death was independent of glutamate treatment i.e. this pattern was detectable in both control and glutamate treated neurons, and was associated with necrotic cell death in our model. We acknowledge that this workflow is not optimal to measure cell death in environments where coordinated cell death may occur. Nevertheless, we did not observe coordinated cell death in neurons seeded at lower densities (<100,000 cells/well; (S2 Fig, S2 Movie)). In addition, neurons plated at higher density often clustered together, leading to segmentation difficulties and fewer neurons accessible for measurement per field of view.

Glutamate excitotoxicity induces mixed responses in neuronal populations, with varying proportions of neurons either surviving or undergoing apoptosis or necrosis depending on insult severity [14,35,36]. In accordance with previous studies, transient glutamate excitation (10 min) led to a mixed population of surviving and apoptotic neurons in our system. Surprisingly, we noted that CGNs exposed to prolonged glutamate treatment (30 min) tended to also die by apoptosis in our system, rather than necrosis. We further pushed the system towards a severe injury model by increasing extracellular Ca^2+^, as overloading of Ca^2+^ is a known mediator of excitotoxic neuronal death [16,18]. As expected, CGNs were more sensitive to glutamate-mediated excitotoxicity with increasing extracellular Ca^2+^ concentrations. This is likely due to excessive Ca^2+^ influx in neurons with elevated extracellular Ca^2+^. Interestingly, only neurons exposed to both high glutamate (300 μM) and Ca^2+^ (2.0 mM) showed increased necrosis.

Neurons deprived of glucose undergo both apoptosis and necrosis following an excitotoxic insult [26,37], while neurons cultured in high glucose are protected against oxygen-glucose deprivation and NMDA-induced excitotoxicity [36,38]. In this study we show that CGNs cultured in low glucose (4 mM) were more susceptible to transient glutamate exposure and underwent increased necrosis compared to neurons cultured in high glucose (15 mM). These results suggest that glucose availability plays an important role in sensitizing neurons to excitotoxicity. Neurons are energy demanding cells, and acute glucose deprivation causes mitochondrial dysfunction, decreased ATP levels, elevation of intracellular calcium levels and cell death [39–41]. In addition, culturing neurons in low glucose (1.7 or 5 mM) significantly inhibited mitochondrial function, reduced cellular ATP levels and production, and sensitized neurons to NMDA-mediated excitotoxicity, increasing necrosis [26,27]. The ability of neurons to both increase the intracellular energetic supply during excitotoxic insults (through substrate uptake and increased ATP production), and to restore energetic homeostasis subsequently, enhances neuronal resistance to glutamate-induced excitotoxicity [36,42]. Our data also suggest that neurons cultured in high glucose retain the ability to undergo controlled apoptotic cell death, potentially reducing the damage to surrounding cells.

At the molecular level, several pathways have been identified to mediate neuronal apoptosis following excitotoxicity, and evidence suggests that these pathways can also interact with each other in excitotoxic injury models. In this study, inhibition of either calpain or PARP activity increased neuronal survival following glutamate excitotoxicity. Indeed, both calpains and PARP-1 activation and subsequent AIF release have been identified as key executioners of excitotoxic apoptosis, which is a largely a caspase-independent process in mature neurons [14,32,33,43,44]. Interestingly, calpains also cleave and inactivate pro-caspase-9 and pro-caspase-3, and inhibit cytochrome-c-induced caspase activation [11,45].

Here, we also showed that inhibition of either AMPK, JNK or mTOR activity protected neurons against glutamate-mediated excitotoxicity, again suggesting convergence of these pathways, or involvement of compensatory mechanisms. Prolonged AMPK activation under energetic stress has been shown to activate the pro-apoptotic BIM protein, and induce apoptosis in neurons [29,30]. BIM expression is also regulated by JNK [46], and previous studies also showed that JNK and p38 kinase are involved in neuronal apoptosis in excitotoxicity and ischemia/reperfusion injury [47–52]. AMPK activation also suppresses protein synthesis through the mTOR pathway [53], and mTOR inactivation inhibits neuronal apoptosis in neurodegenerative disorders [54].

Together, these results indicate the convergence of different cellular pathways, culminating in neuronal cell death. Because inhibition of no single pathway completely inhibited apoptosis in our system, this also suggests redundancy or compensatory mechanisms in these apoptotic pathways. The HCS assay developed here can help to dissect the most important pathways involved in excitotoxic cell death, their cross talk, and the combinatorial effect of inhibiting different components of these pathways.

In conclusion, we have developed a HCS cell-based *in vitro* imaging and analysis application, which is a valuable tool to investigate the contribution of external factors and intrinsic components in the neuronal survival and cell death pathways following glutamate excitotoxicity.

## Supporting information

S1 Fig Segmentation and isolation of PI stained neurons in real time high content screening experiment. Mouse cerebellar granular neurons cultured in 96 well plates were stained with PI (250 ng/ml) in conditioned media. A) Representative image of a field of view acquired by HCS, showing masks for PI-stained nuclei (green outlines). Scale bar: 40 μm. (B) Zoomed area (white box in A) showing PI intensity, object masks and segmented object areas from CellProfiler. Green outlines represent objects that were measured for further analysis while red outlines were discarded due to size restrictions. Scale bar: 10 μm.
(TIF)

S2 Fig Coordinated cell death was identified in neurons seeded at high density. CGNs were cultured *in vitro* for 7 days before stimulating with glutamate. To monitor coordinated cell death, PI intensity was quantified using ImageJ in the regions highlighted. Quantification and time-lapse image series of PI staining in neurons seeded at A) 100,000 cells/well and B) 50,000 cells/well. A) An increase in PI intensity was observed to propagate wave-like from the bottom right corner of the field of view to the upper left corner. A rapid increase in excitotoxic cell death was observed in region 2, while a similar but delayed increase was measured in region 1, indicating wave progression. B) Although there was an increase in visual PI stained nuclei, quantified PI intensity is similar and time-independent in highlighted regions 1 and 2. No wave progression was observed in this field of view.
(TIF)

S3 Fig Cytosolic calcium levels during glutamate exposure are dependent on extracellular calcium. CGNs were stimulated with glutamate/glycine (100/10 μM) for 10 min in the presence of varying concentrations of extracellular Ca^2+^, as indicated. A) Representative traces of Fluo-4-AM fluorescence intensity (Ca^2+^ indicator) during transient glutamate excitation at various Ca^2+^ concentrations. B) Quantification of the glutamate-induced Ca^2+^ increase as area under the Fluo-4 fluorescence intensity curve in glutamate treated neurons in 0.05, 0.1, 0.5, 1.5 and 2 mM extracellular Ca^2+^ (n = 15, 12, 19, 31 and 23, respectively). Data presented as mean (SEM). *p < 0.01 difference between area under the curve compared to neurons in 0.05 mM Ca^2+^.
(TIF)

S1 Movie Representative movie showing coordinated cell death in neurons. CGNs were cultured *in vitro* for 7 days before stimulating with glutamate. To visualize coordinated cell death, time-lapse imaged series of PI staining was monitored in neurons seeded at 100,000 cells/well. Coordinated cell death in neurons showing wave-like propagation.
(MOV)

S2 Movie Representative movie showing non-coordinated cell death in neurons. CGNs were cultured *in vitro* for 7 days before stimulating with glutamate. To visualize non-coordinated cell death, time-lapse imaged series of PI staining was monitored in neurons seeded at 50,000 cells/well. Non-coordinated cell death was observed in neurons.
(MOV)
