# Supplementary figures and images for: Defining external factors that determine neuronal survival, apoptosis and necrosis during excitotoxic injury using a high content screening imaging platform
Source: PLoS One. 2017 Nov 16;12(11):e0188343. doi: 10.1371/journal.pone.0188343 (PMC5690623; doi:10.1371/journal.pone.0188343)

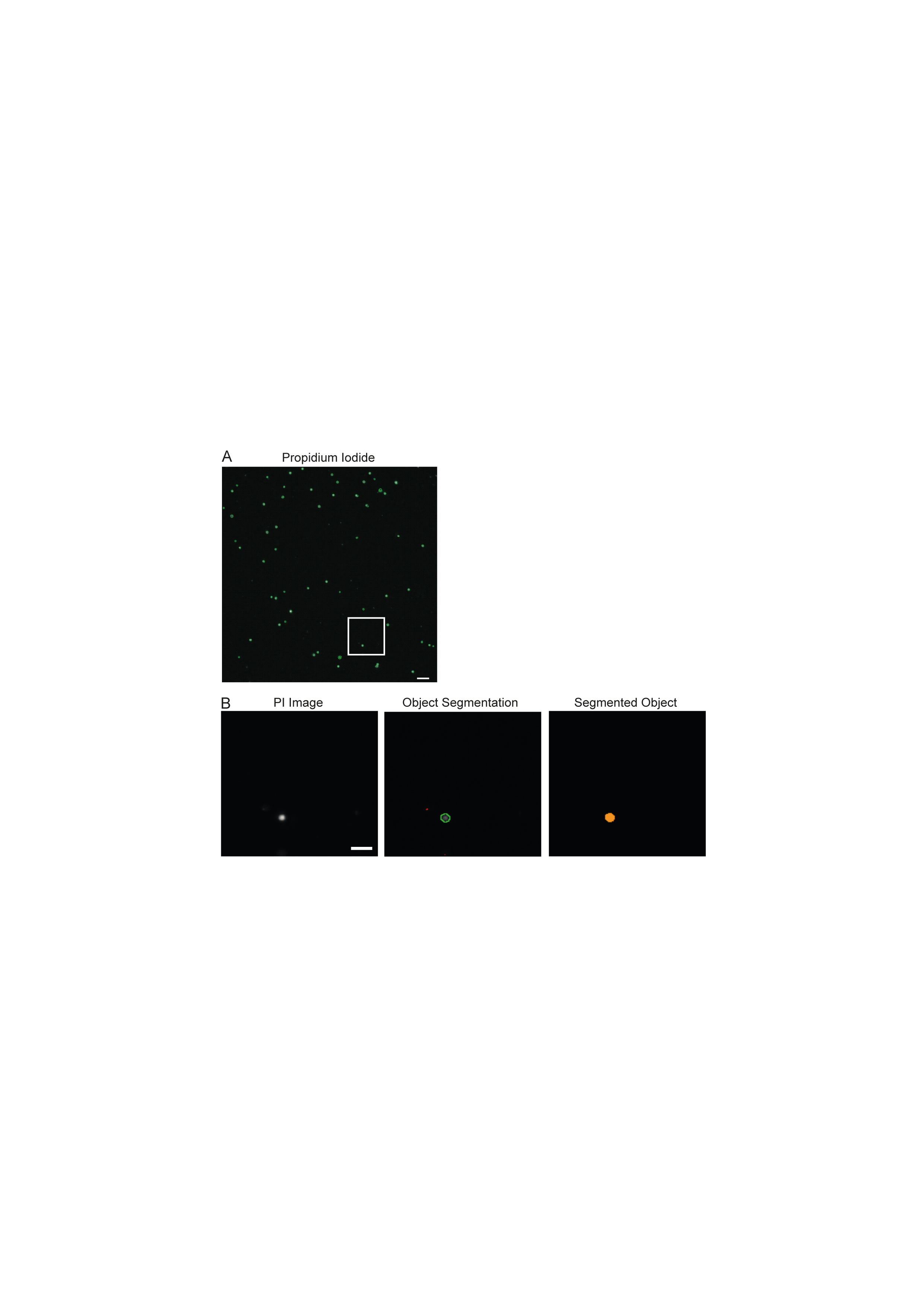

Supplement: S1 Fig — Mouse cerebellar granular neurons cultured in 96 well plates were stained with PI (250 ng/ml) in conditioned media. A) Representative image of a field of view acquired by HCS, showing masks for PI-stained nuclei (green outlines). Scale bar: 40 μm. (B) Zoomed area (white box in A) showing PI intensity, object masks and segmented object areas from CellProfiler. Green outlines represent objects that were measured for further analysis while red outlines were discarded due to size restrictions. Scale bar: 10 μm. (TIF) [file pone.0188343.s001.tif]

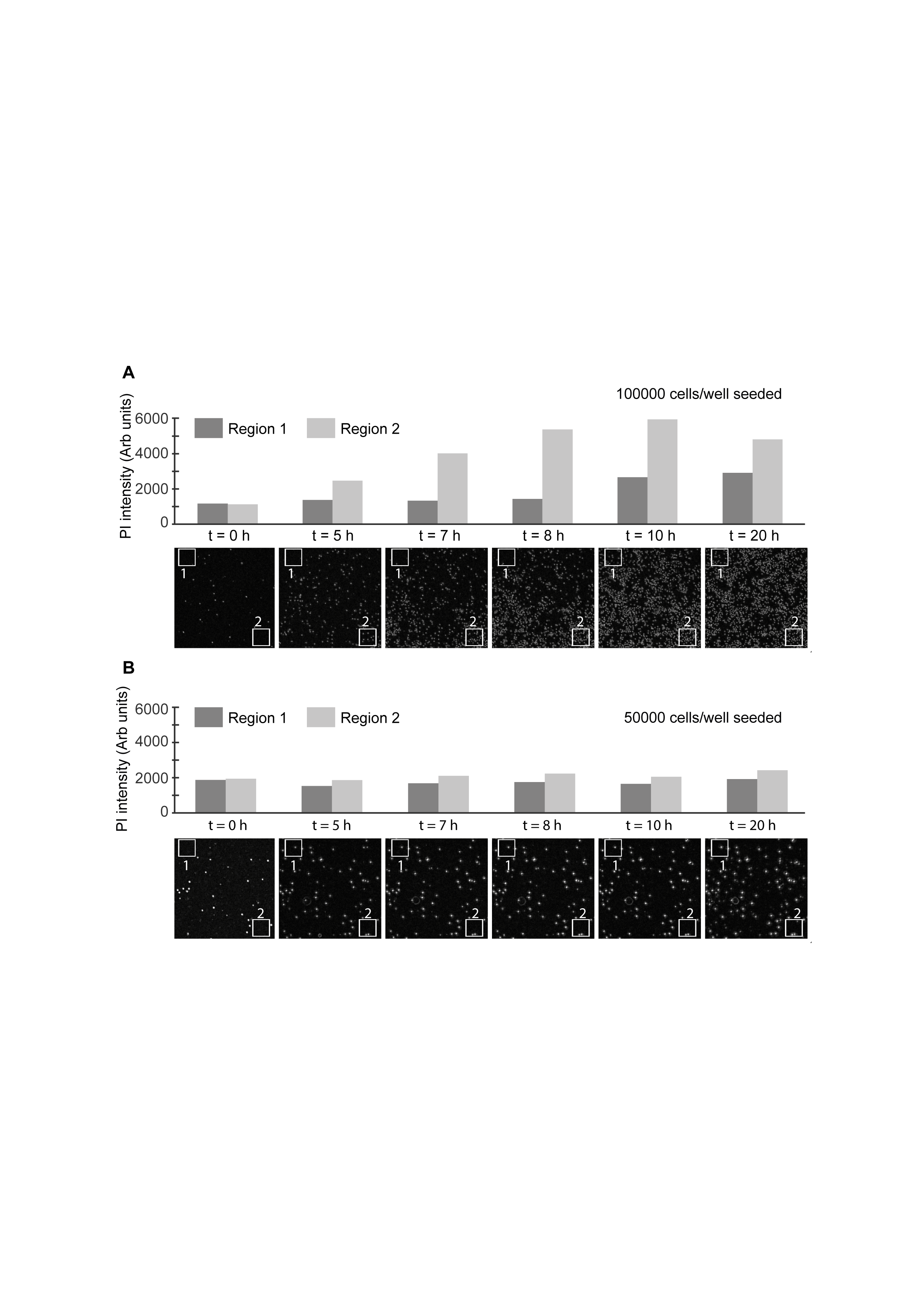

Supplement: S2 Fig — CGNs were cultured in vitro for 7 days before stimulating with glutamate. To monitor coordinated cell death, PI intensity was quantified using ImageJ in the regions highlighted. Quantification and time-lapse image series of PI staining in neurons seeded at A) 100,000 cells/well and B) 50,000 cells/well. A) An increase in PI intensity was observed to propagate wave-like from the bottom right corner of the field of view to the upper left corner. A rapid increase in excitotoxic cell death was observed in region 2, while a similar but delayed increase was measured in region 1, indicating wave progression. B) Although there was an increase in visual PI stained nuclei, quantified PI intensity is similar and time-independent in highlighted regions 1 and 2. No wave progression was observed in this field of view. (TIF) [file pone.0188343.s002.tif]

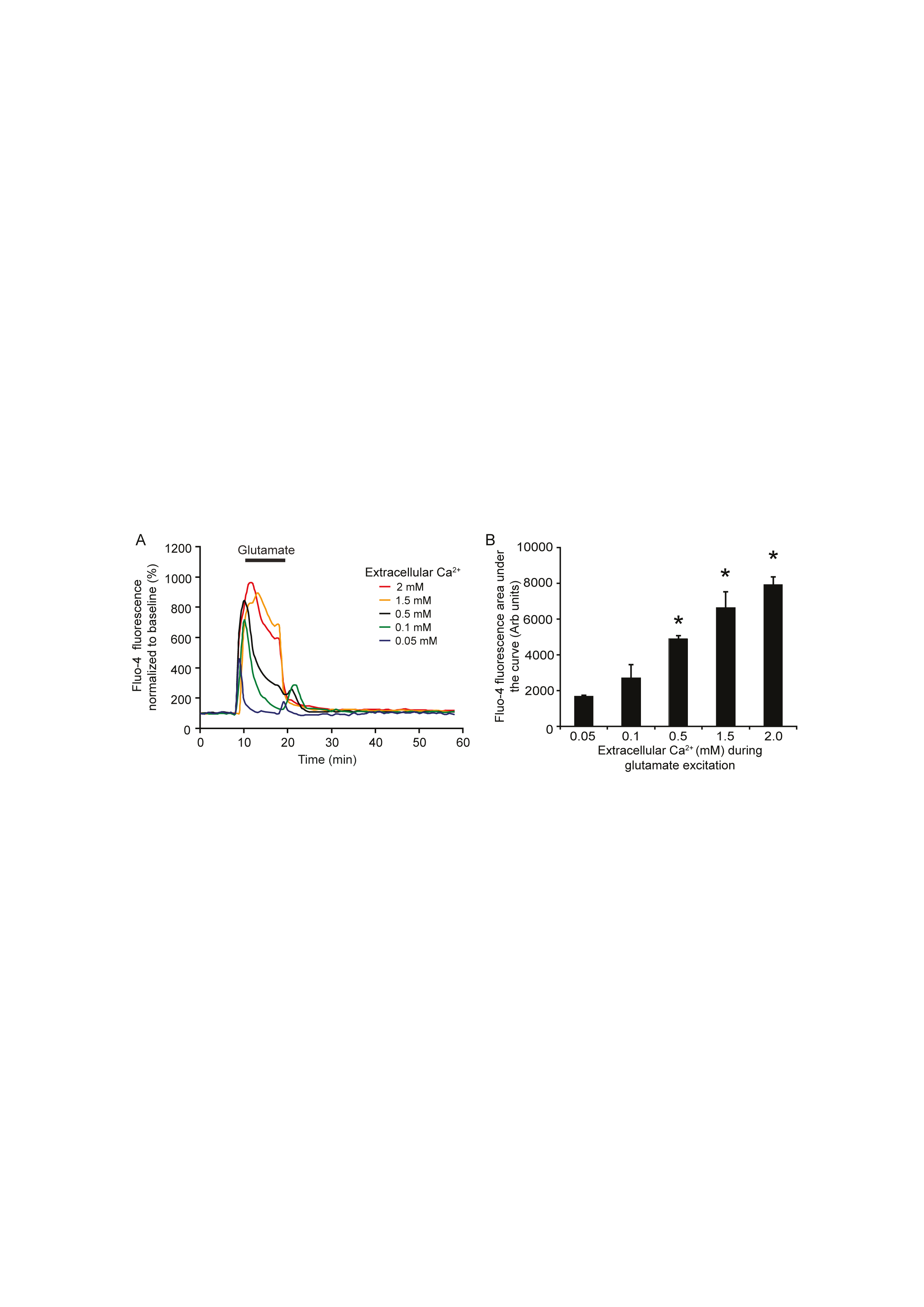

Supplement: S3 Fig — CGNs were stimulated with glutamate/glycine (100/10 μM) for 10 min in the presence of varying concentrations of extracellular Ca2+, as indicated. A) Representative traces of Fluo-4-AM fluorescence intensity (Ca2+ indicator) during transient glutamate excitation at various Ca2+ concentrations. B) Quantification of the glutamate-induced Ca2+ increase as area under the Fluo-4 fluorescence intensity curve in glutamate treated neurons in 0.05, 0.1, 0.5, 1.5 and 2 mM extracellular Ca2+ (n = 15, 12, 19, 31 and 23, respectively). Data presented as mean (SEM). *p < 0.01 difference between area under the curve compared to neurons in 0.05 mM Ca2+. (TIF) [file pone.0188343.s003.tif]
